# Factors that influence the employability skills of vocational school students: A Systematic Review

**DOI:** 10.12688/f1000research.164629.1

**Published:** 2025-06-16

**Authors:** Qori Agussuryani Puji Hartini, Wiyanto Wiyanto, Sudarmin Sudarmin, Woro Sumarni

**Affiliations:** 1 Department of Natural Science Education, Faculty of Mathematics and Natural Sciences, Universitas Negeri Semarang, Semarang, Central Java, Indonesia; 2 Department of Elementary School Education, Faculty of Tarbiyah and Teacher Training, Universitas Sains Alqur an, Wonosobo, Central Java, Indonesia

**Keywords:** Employability skills, Influencing Factors, PRISMA analysis, Vocational Education, Vocational School Students

## Abstract

Employability skills of vocational high school students are needed to prepare for a reliable, professional, and globally competitive world of work. The poor work skills of vocational high school students are influenced by many factors. This study analyzes the research trends and factors that influence vocational high employability. Type of literature study using a Preferred Reporting Items for Systematic Reviews and Meta-Analyses (PRISMA). Data collection for 2020-2024 Work skills based on a search of Google Scholar, Scopus, Science Direct, and Web of Science databases was conducted from October 25 to 31, 2024. VOSviewer application to analyze research trends. Search using keyword employability skills and vocational high school students. The selection process was conducted by inviting three authors to review articles. If an article does not meet the criteria, the solution is to invite other reviewers or experts to help complete it. Data analysis techniques include an identification stage with records from the database and continued filtering of data that meets the criteria and does not. Filtering stage with feasibility assessment Study Stage in Article Review. The results of the study identified 1016 articles, 311 articles, 39 articles, and 96 factors influencing work skills. Six dominant factors determine work skills: creativity (FI_1), communication skills (FI_2), adaptability (FI_3), problem-solving (FI_4), critical thinking (FI_5), and self-confidence (FI_6). The determining factors of work skills internationally are more dominant in self-confidence, adaptability, and communication skills, while in Indonesia, they are communication skills and creativity. A limitation of the data search process is the use of incomplete keywords. The contribution of this study is that six determining factors were used as considerations for the qualifications of student graduates in contextual and meaningful vocational learning.

## Introduction

Employability skills support vocational school students’ careers and work readiness Career support is in the form of integrating work skills into vocational learning. ^1, 2^ Factors that influence students’ low employability skills include teachers’ inability to innovate and design meaningful vocational learning. Students’ employability skills have not been able to produce graduates who are ready to work and globally competitive. ^3^ Empowering employability skills can help students solve problems, adapt to the environment, cultivate social attitudes, Information Technology (IT) skills, language literacy, personal abilities, self-leadership skills, and communication skills. ^4, 5^ Employability skills are an important requirement before graduating from vocational school so that they are ready to compete in the world of work. ^6, 7^

The importance of Employability Skills as skills that enable students to get a job after graduating includes personal and interpersonal skills, attitudes, habits, and behavior. Multifactorial problems originating from students, teachers, and the vocational education system in Indonesia influence students’ low employability. The Merdeka Curriculum for vocational schools in intracurricular learning is divided into general and vocational groups. The learning process is still theoretical and does not fully integrate work skills. The world of work requires vocational school graduates to be competent and professional, but employability skills have not been fully realized and developed in vocational learning.

The problem of unemployment in Indonesia, based on records from the Central Statistics Agency (BPS), shows that the number of open unemployed nationally in February 2024 will reach 7.2 million people. Unemployment data for vocational school graduates in the last 3 years is 11.13 million people (2021); 9.42 million people (2022); and 9.31 million people (2024). Unemployment data was also confirmed through the vocational school alumni tracker in Wonosobo Regency, showing that 43% of vocational school graduates have worked according to their competencies, and the remaining 67% of vocational school graduates are not ready to compete and have work skills. High unemployment rates in developing countries are not only resolved with an economic approach but must be balanced with increased competence according to industry needs. ^8, 9^ Vocational schools, as educational institutions, must be able to prepare graduates for the world of work and industry (DUDI) by professionally mastering technology and soft skills. ^10^ A comparison of international and national research on vocational learning revealed differences in job skills. In general, Indonesia has only curriculum content and targeted material, as proven in the national assessment; concept understanding tests are still prioritized, not work skill readiness.

Employability skills in vocational learning can help develop appropriate learning strategies to equip graduates. Students can acquire knowledge that is directly related to real life (contextual), utilizing the environment, and reconstructing traditions, culture, and local potential, which can be used as a source of meaningful learning. A vocational learning approach based on local wisdom can be used to reconstruct a community’s original science into scientific knowledge through work skills. The core competency of vocational graduates is their employability. The more complex the learning resources in society, the more opportunities there are to develop vocational school students’ employability skills. Work-ready competencies are very important to develop by understanding students’ employability skills. The problem of employability skills is very complex in analyzing dominant factors, so the research objectives are to analyze research trends in the employability skills of vocational school students and factors that influence the employability skills of vocational school students.

## Methods

**Figure 1.**
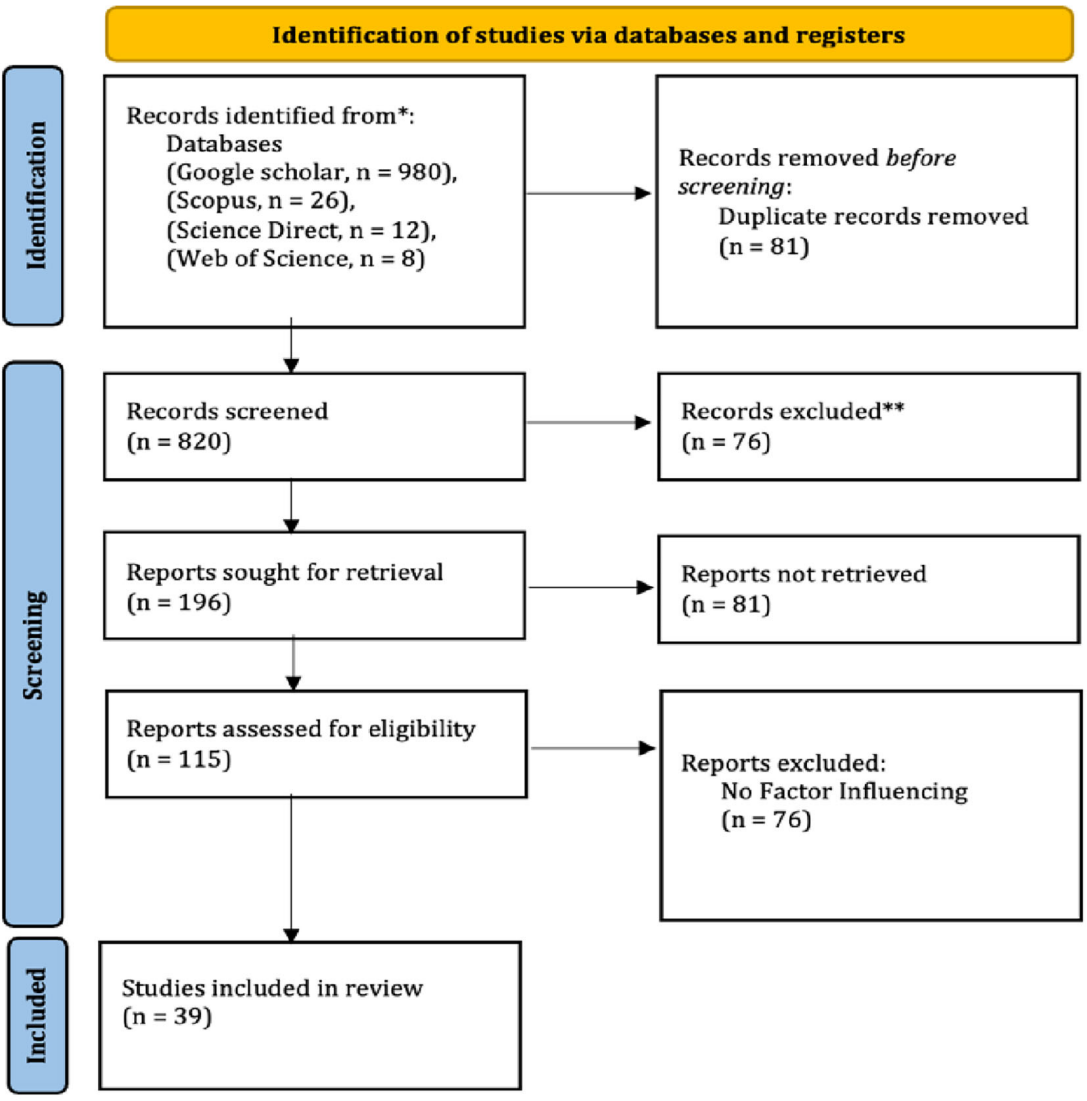
PRISMA flowchart for employability skills.

The literature review used the Preferred Reporting Items for Systematic Reviews and Meta-Analyses (PRISMA) systematic review approach ( Figure 1). ^11^ Research limitations used the keywords “employability skills” AND “vocational school students.” Data sources were based on articles published and indexed by Google Scholar, Scopus, Science Direct, Web of science in 2020-2024 (last five years), and then analyzed using the VOSviewer application. The data search was conducted from October 25 to October 31, 2024. Eligibility criteria were determined by determining inclusion and exclusion using the PESTLE Analysis are presented in Table 1.

**Table 1.** Inclusion and Exclusion criteria.

Component	Inclusion criteria	Exclusion criteria
POLITICAL FACTORS *Elections, change of government leadership, potential policy changes, rule of law, etc.*	Articles can be accessed in full text including title, abstract, keywords and complete content	Does not have full text
ECONOMIC FACTORS *Economic growth or stagnation, interest rates, exchange rates, inflation, unemployment, etc.*	Published in the period 2020-2024	Article publication before 2020
SOCIAL FACTORS *Population and demographic changes, health conditions, level of education, social mobility, social attitudes, religious beliefs, socio-cultural changes, etc.*	• Article category “research article” • Articles use english language	• Articles that use languages other than English • Articles in the form of literature reviews, book reviews, meta-analyses, or articles on the development of measuring instruments
TECHNOLOGICAL FACTORS *Changes in the availability or price of new technologies, technological infrastructure, potential changes in technological standards, etc.*	Articles discussing the measurement of students' employability skills	Does not include vocational school learning that measures students' employability skills
LEGAL FACTORS *Labor laws, relevant court cases, employment regulations, etc.*	Government policy on work skills needed by vocational school graduates	Does not include policies on employability skills of vocational school students
ENVIRONMENTAL FACTORS *Climate, weather, energy consumption regulations, etc.*	• The article category discusses employability skills at the vocational school level • The article contains learning for vocational school age	Duplicate or multiple articles

This study only uses English-language journals and does not take data from books, proceedings, erratum, and others.

In the journal selection process, the researcher invited three authors to review the articles obtained; if there was a discrepancy in determining the article based on the criteria, the solution was to invite another reviewer or expert to help solve the problem.

Identification begins with a search of Google Scholar, Scopus, Science Direct, and Web of Science databases. Before the screening process, a duplication check was carried out. Next, title and abstract screening was carried out. Articles meeting the inclusion criteria will be checked for full text.

The screening process uses automatic tools, namely rayan.ai, which aims to help screen and analyze the articles found.

Journal grouping was based on the criteria for the most dominant employability skills determinants of each article.

Information on the determinants of vocational high school students’ employability skills is still considered to provide insufficient information, which is done by reviewing other references in the form of vocational high school curriculum policies related to indicators of graduate success.

## Results

The search resulted in 980 articles from the Google Scholar database and 36 articles from Scopus, Science Direct, and Web of Science. The initial stage is to remove duplicate data, non-article data such as books, proceedings, book chapters, data that are not in English, and the year of publication before 2020 (N = 820). After the filtering stage, 196 articles remained. The filtering stage removed non-full-text article data, not at the vocational school level, and did not include the inclusion criteria (N = 76). A total of 115 articles were included in the filtering stage. Furthermore, based on the eligibility of articles on factors that influence the work skills of vocational high school students, keywords, article titles, and meeting the eligibility criteria for review, 39 articles were produced.

The results of the systematic review show Employability Skills as a solution for training students in scientific work and being directly involved in the vocational learning process and producing products. Vocational learning activities to develop work skills that are not focused on one solution to the problems they face but have many alternative answers to solve problems based on work skills indicators. The importance of workability in vocational learning, the potential for workability, the factors that influence it, and the relationship between workability components and other indicators. The publication data findings for the last five years (2020-2024) for research trends are presented in Table 2.

**Table 2.** PRISMA results-based publication data source.

Year	Identification		Screening		Included	
	A	B	A	B	A	B
2020 (1st)	200	12	59	10	6	3
2021 (2nd)	200	3	69	1	9	0
2022 (3rd)	200	9	58	8	6	1
2023 (4th)	180	8	50	8	6	1
2024 (5th)	200	4	41	7	6	1
Total	980	36	277	34	33	6
	1016		311		39	

A: Google Scholar, B: Scopus.

**Figure 2.**
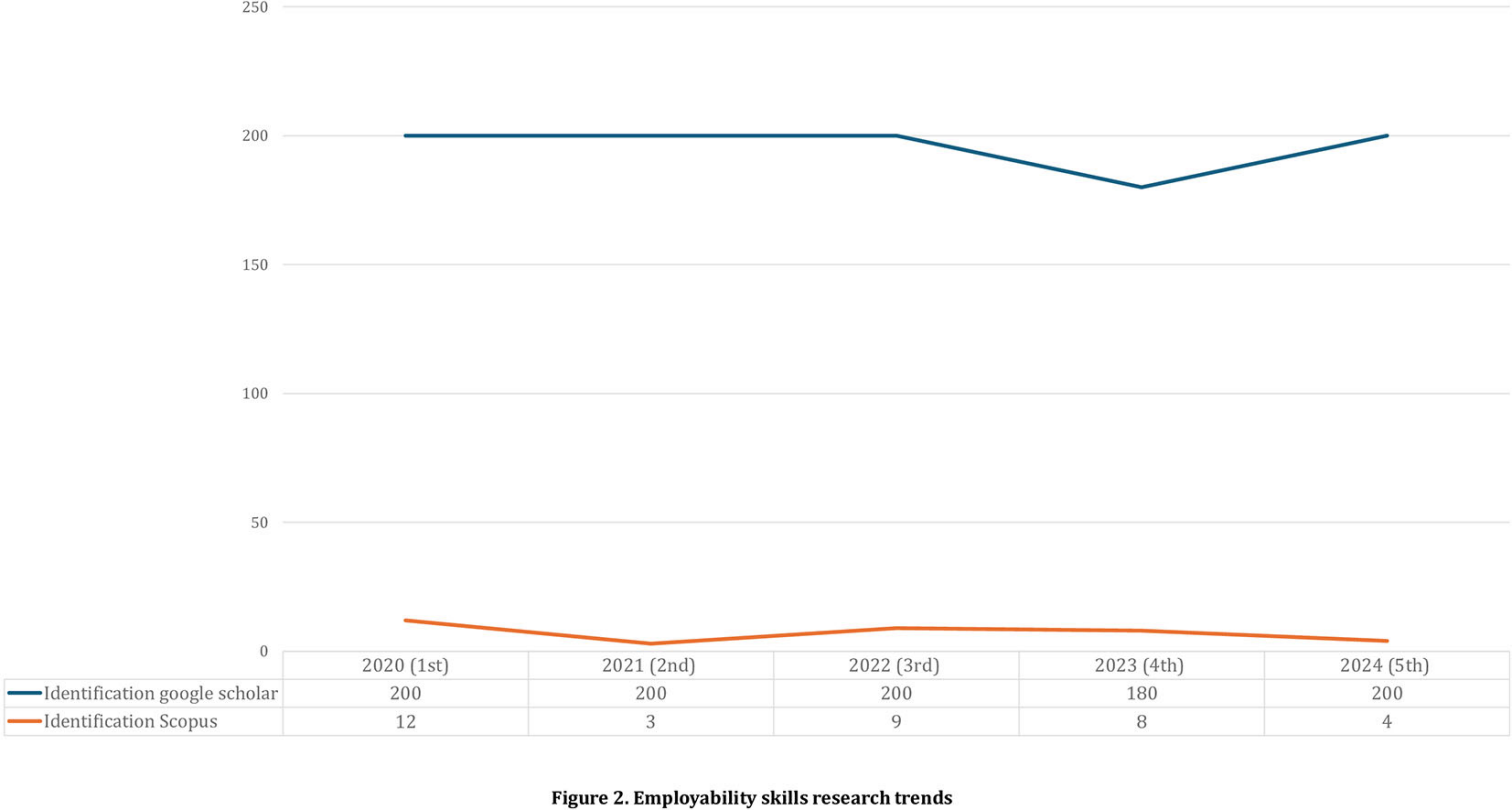
Employability skills research trends.

Table 2 PRISMA analysis results totaling 39 articles in reputable international journal categories indexed by Scopus and Google Scholar based on research inclusion criteria. Employability Skills research trends based on year of publication in the last 5 years are presented in Figure 2.

**Figure 3.**
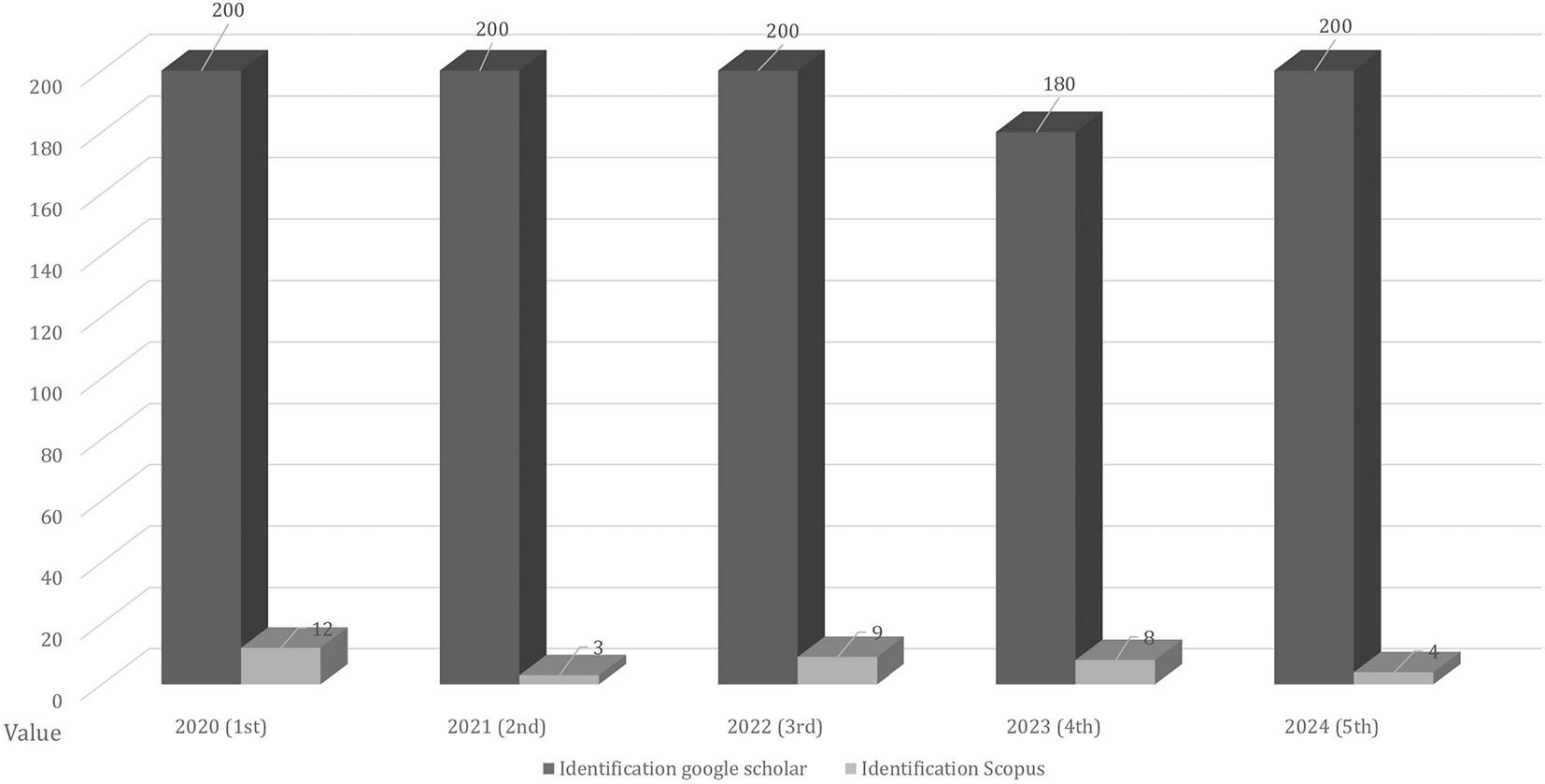
Scopus and google scholar indexed publications.

Figure 2 shows that the publication trend over the last five years has fluctuated because of the many needs for employability skills that are considered necessary to prepare work-ready graduates and adapt to global needs. By 2023, there will be a significant decrease compared to the previous year. Publications indexed by Scopus and Google Scholar are presented in Figure 3.

**Figure 4.**
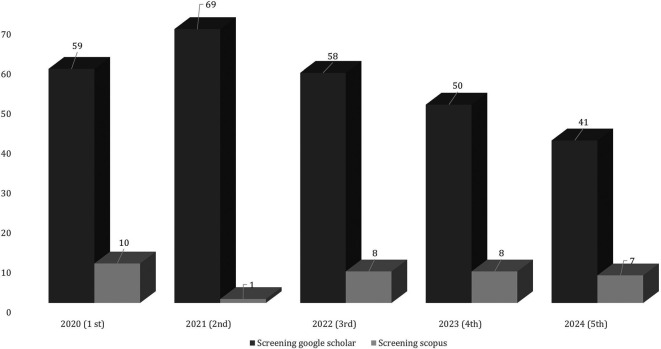
Screening of employability skills publication data.

Figure 3 shows international journal publications indexed by Scopus and Google Scholar based on research inclusion criteria to maintain the validity and authenticity of the data sources. A reputable international journal determined that the results and quality of the research can be accounted for by research ethics. The screening data after identifying the publications are shown in Figure 4.

**Figure 5.**
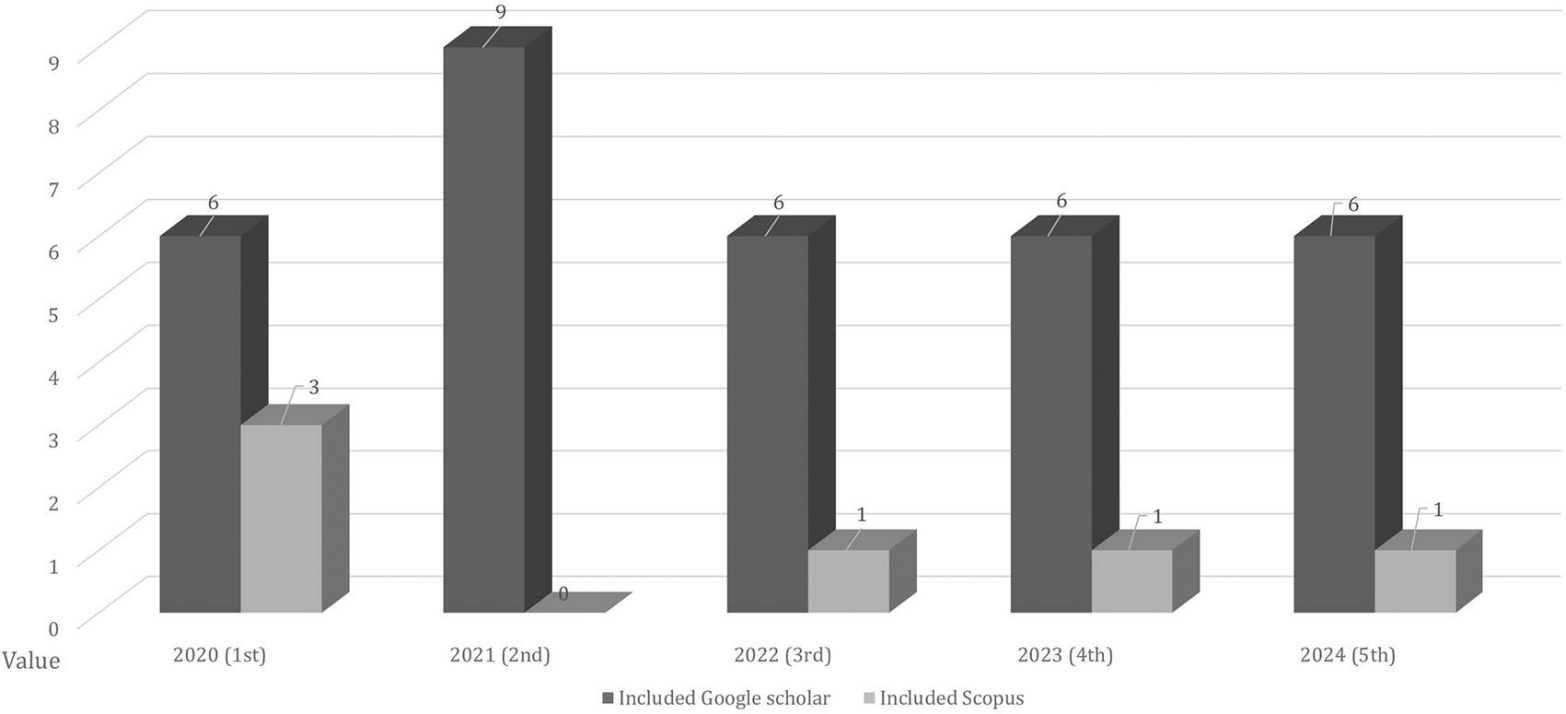
Based on included data.

Figure 4 data screening shows several duplicate publications, the type of publication is not in a journal, and does not match the inclusion and scope of the research. In 2021, the most publications were 69 indexed by Google Scholar and in 2020 there were 59 articles indexed by Scopus. The next step in determining research data inclusion is presented in Figure 5.

**Figure 6.**
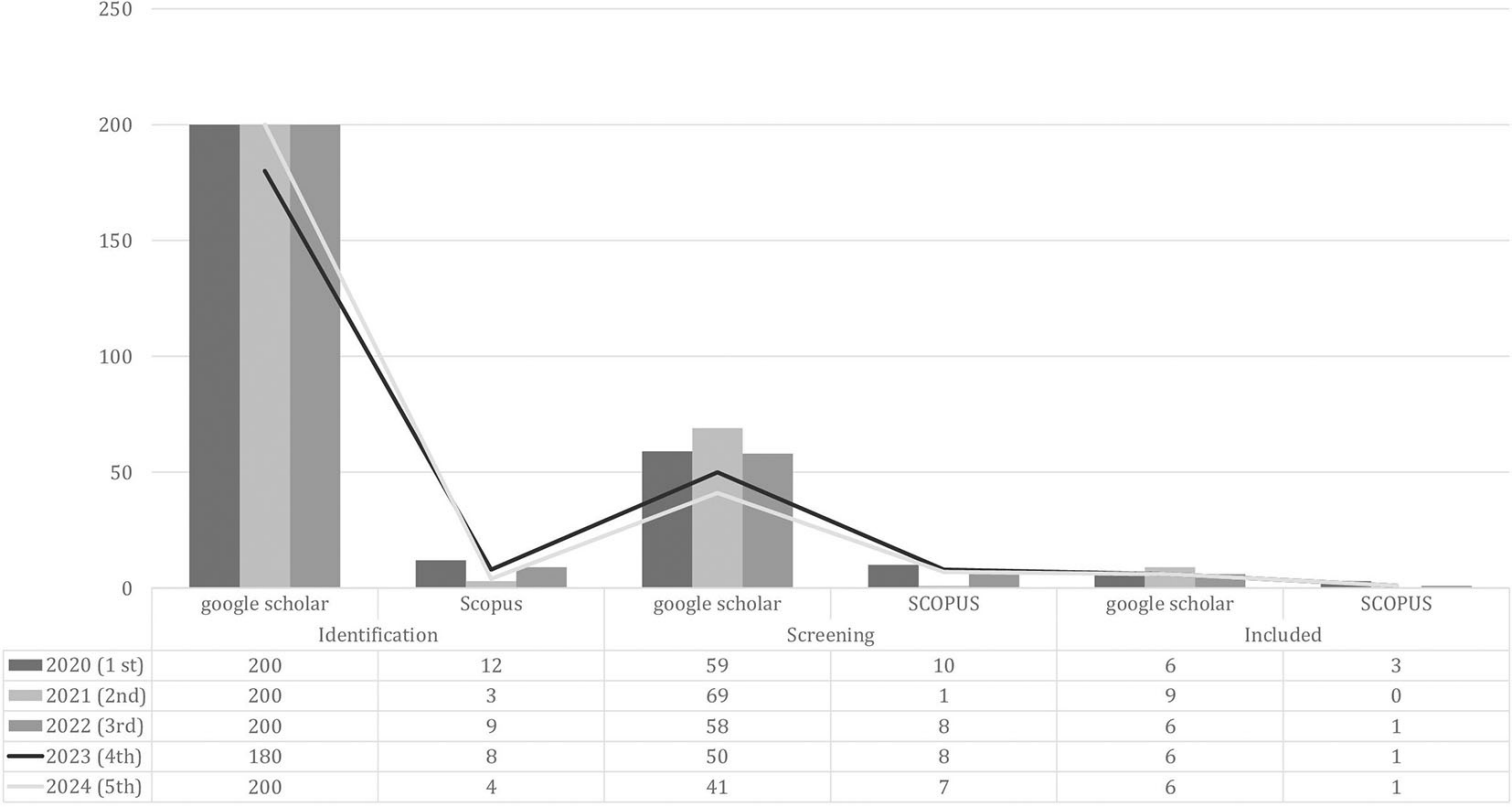
PRISMA Analysis data distribution based on determining factors of student employability skills.

The research inclusions in Figure 5 show data that are in accordance with the research objectives, so that these data determine the results of the PRISMA analysis. 2021 received the most inclusion data compared with the other years. The overall distribution of data at each stage is shown in Figure 6.

**Figure 7.**
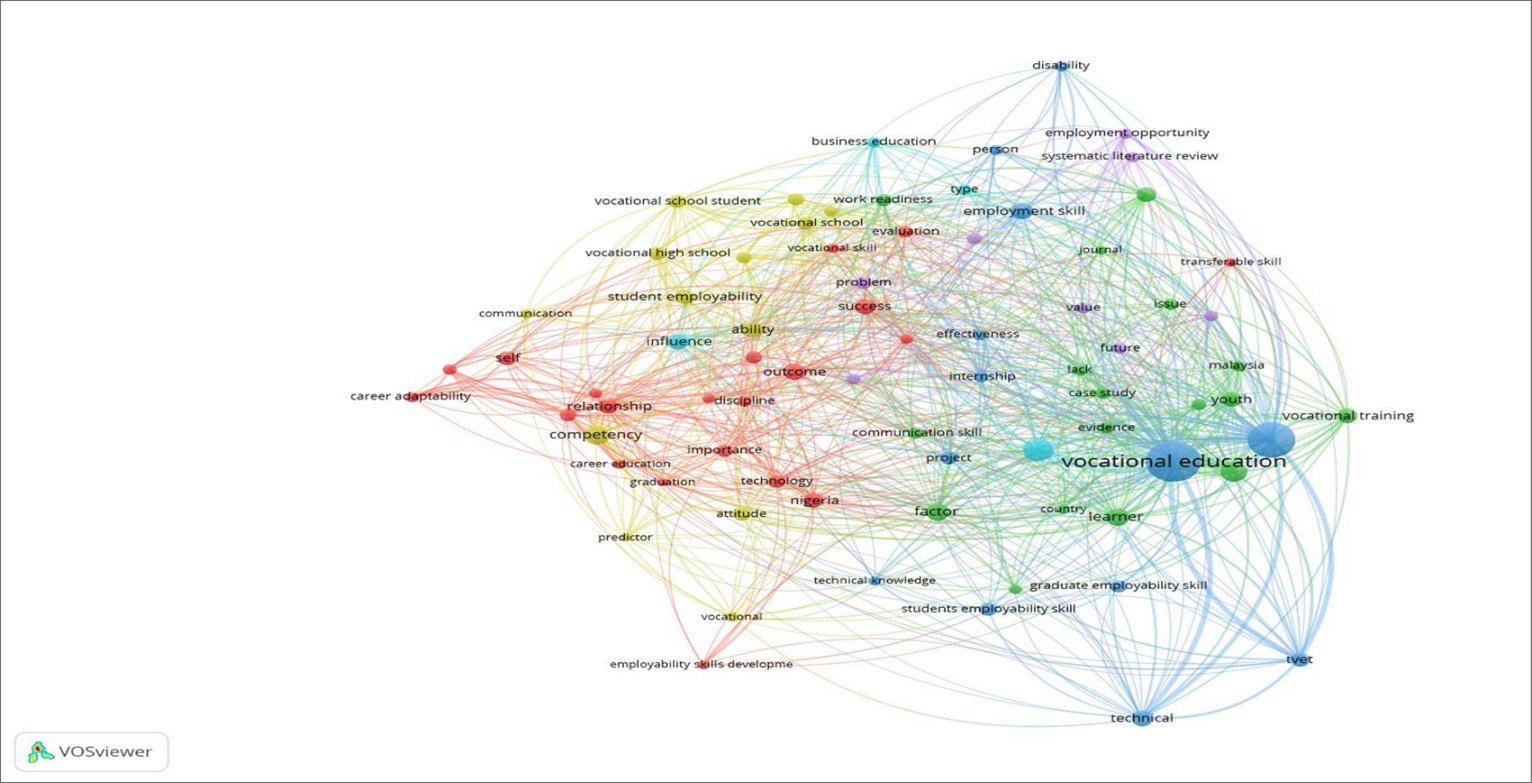
Visualization of vocational learning research trends.

The distribution of the PRISMA data in Figure 6 shows the consistency and reliability of the objective data. Based on the data from the identification process up to the inclusion data, the numbers became smaller, meaning that step-by-step has been carried out according to research principles. Trends in employability skills research using the VOSviewer application with keywords for the last five years (2020-2024) based on Publish or Perish searches are presented in Figure 7.

The visualization results in Figure 7 of the research trends for the last five years are vocational education, training, acquisition, and employment skills, which are shown in the largest circle. The color in each visualization shows the same cluster in the study. The closer the distance between studies, the stronger the connection, and the stronger the citations between journals are represented by lines. Vocational education does not fully refer to employment skills and is more dominant in technological vocational education training; therefore, it is necessary to further study employability skills factors. Furthermore, Vocational school students do not yet have a research network connection with employability skills, meaning that this study has a high degree of novelty. Next, the influencing factors (FI) to map the largest determining factors that can be used as indicators of employability skills are presented in Table 3.

**Table 3.** Data included based on PRISMA analysis.

Author	Country	Number Factor Influences (N FI)
39	14	96

**Figure 8.**
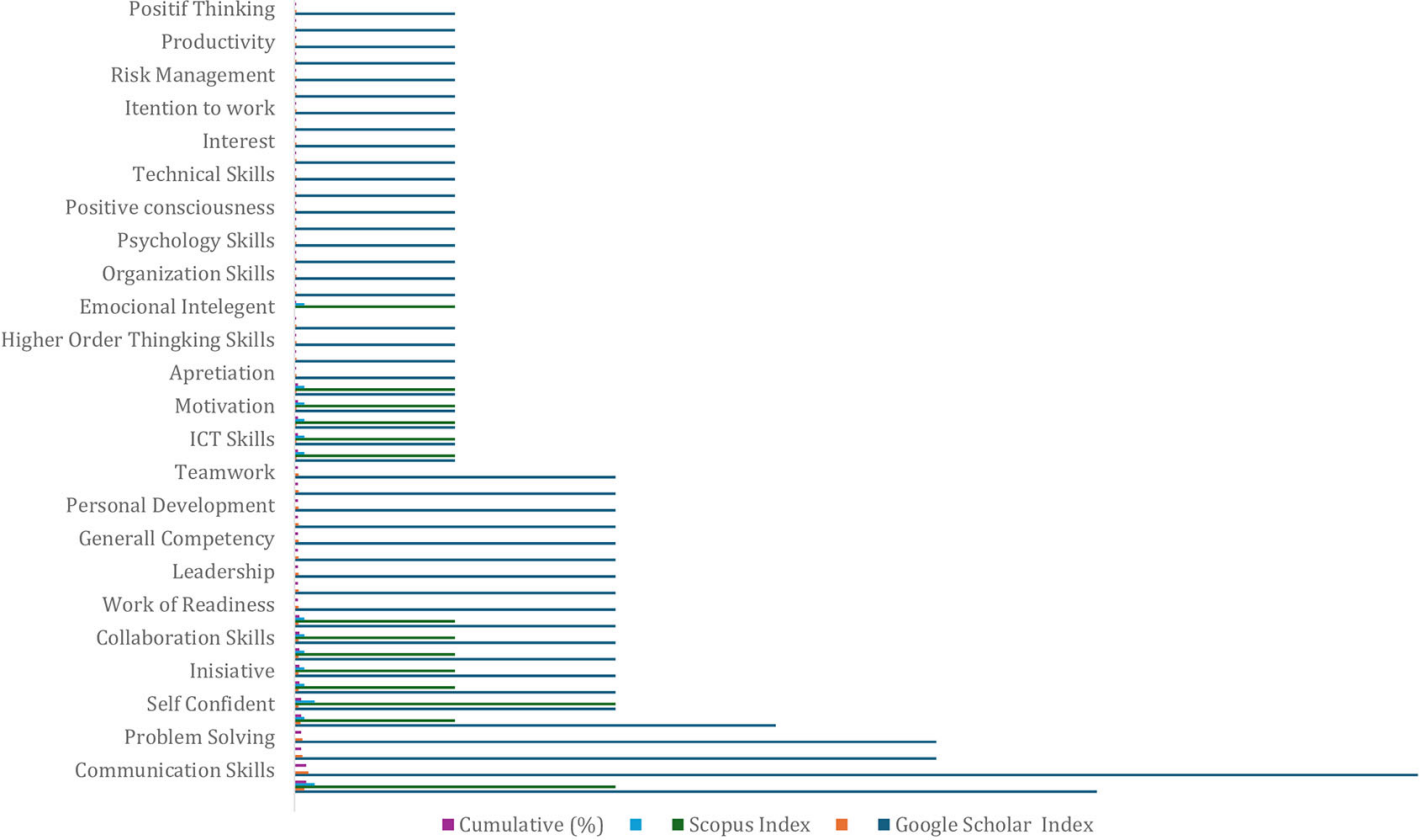
Results 96 factors determining employability skills of vocational school students.

Table 3 includes data from 39 scientific articles indexed using Google Scholar and Scopus. Over the last five years (2020-2024) there have been 80 factors determining employability skills sourced from Google Scholar indexed journals and 16 employability skills factors sourced from Scopus indexed journals; thus, a total of 96 determining factors are presented in Figure 8.

There were 96 determining factors resulting from a literature review using PRISMA based on the percentage of all existing factors, so dominant contribution mapping was carried out using six indicators of determining factors. The results for the six employability skill indicators are presented in Table 4.

**Table 4.** Six factor influencing employability skills based on literature review.

Factor influencing	Google scholar index		Scopus index	
	∑	%	∑	%
Creativity	5	20	2	40
Communication Skills	7	28	0	0
Adaptability	4	16	0	0
Problem Solving	4	16	0	0
Critical Thinking skills	3	12	1	20
Self Confident	2	8	2	40
Total	25	100	5	100

Table 4 shows 39 articles, 17 of which originated from Indonesia and 22 from outside Indonesia (international). Table 5 presents trends in vocational learning research that examine global employability skills from international sources.

**Table 5.** Employability skills research by country of origin.

No	Country	N Article
1	Indonesia	17
2	China	2
3	Croatia	1
4	India	2
5	Malaysia	6
6	Nepal	1
7	Nigeria	2
8	Philippines	2
9	Romania	1
10	Russia	1
11	South Africa	1
12	Spain	1
13	Switzerland	1
14	Tanzania	1
	Total	39

Table 5 shows research trends based on country of origin for 14 countries in the last 5 years. In international research (56%) and Indonesia (44%). Furthermore, in Figure 8 there are 41 factors determining employability skills which come from global research and the 3 largest employability skills factors are self-confidence, adaptability, and communication skills. Research on employability skills in Indonesia is broadly explained based on Table 6.

**Table 6.** Factors of work skills in Indonesia.

No	Factor Influencing (FI)	N FI
1	Communication Skills	4
2	Creativity	4
3	Critical Thinking skills	3
4	General Competency	3
5	Work of Readiness	3
6	Atitude	2
7	Collaboration Skills	2
8	Decisions Making	2
9	ICT Skills	2
10	Inovation	2
11	Konowledge	2
12	Problem Solving	2
13	Self eficacy	2
14	Skills Development	2
15	Adaptability	1
16	Apretiation	1
17	Career Decisions	1
18	Entrepeneur Skills	1
19	Higher order Thinking Skills	1
20	Inisiative	1
21	Interpersonal Skills	1
22	Language Skills	1
23	Leadership	1
24	Morality	1
25	Personal Development	1
26	Personal Skills	1
27	Positive Thinking	1
28	Psycology Skills	1
29	Responsibility	1
30	Risk Decision	1
31	Self Regulation	1
32	Teamwork	1
33	Time Management	1
34	Work Habituation	1
	Total	55

**Figure 9.**
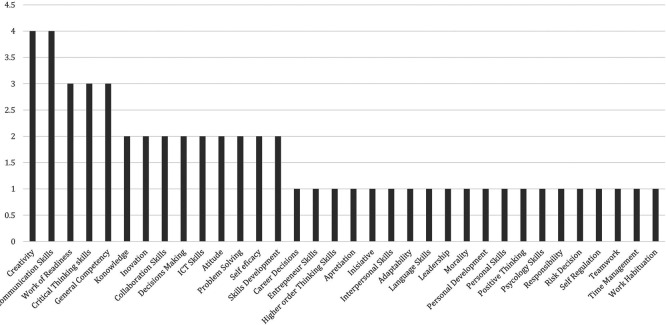
Factors of employability skills in Indonesia.

Table 6 shows 55 work skill factors in Indonesia. Next, to identify the most dominant factors, there are 3 work skill factors, namely communication skills, creativity, and critical thinking skills, presented in Figure 9.

The data revealed differences in work skill factors in vocational learning internationally and in Indonesia. The most dominant determining factors differ internationally: first, self-confidence equips individuals with confidence and self-confidence in completing tasks or goals; second, adaptability is used to adjust to new conditions in the work environment; third, communication skills equip students with how to communicate effectively as a form of interaction skills with colleagues and teams. According to research on employability skills in Indonesia, the biggest determining factors are communication skills and creativity, which are used to produce new ideas and work. The biggest factors found both internationally and nationally relate to 21st-century skill needs.

## Discussion

**Figure 10.**
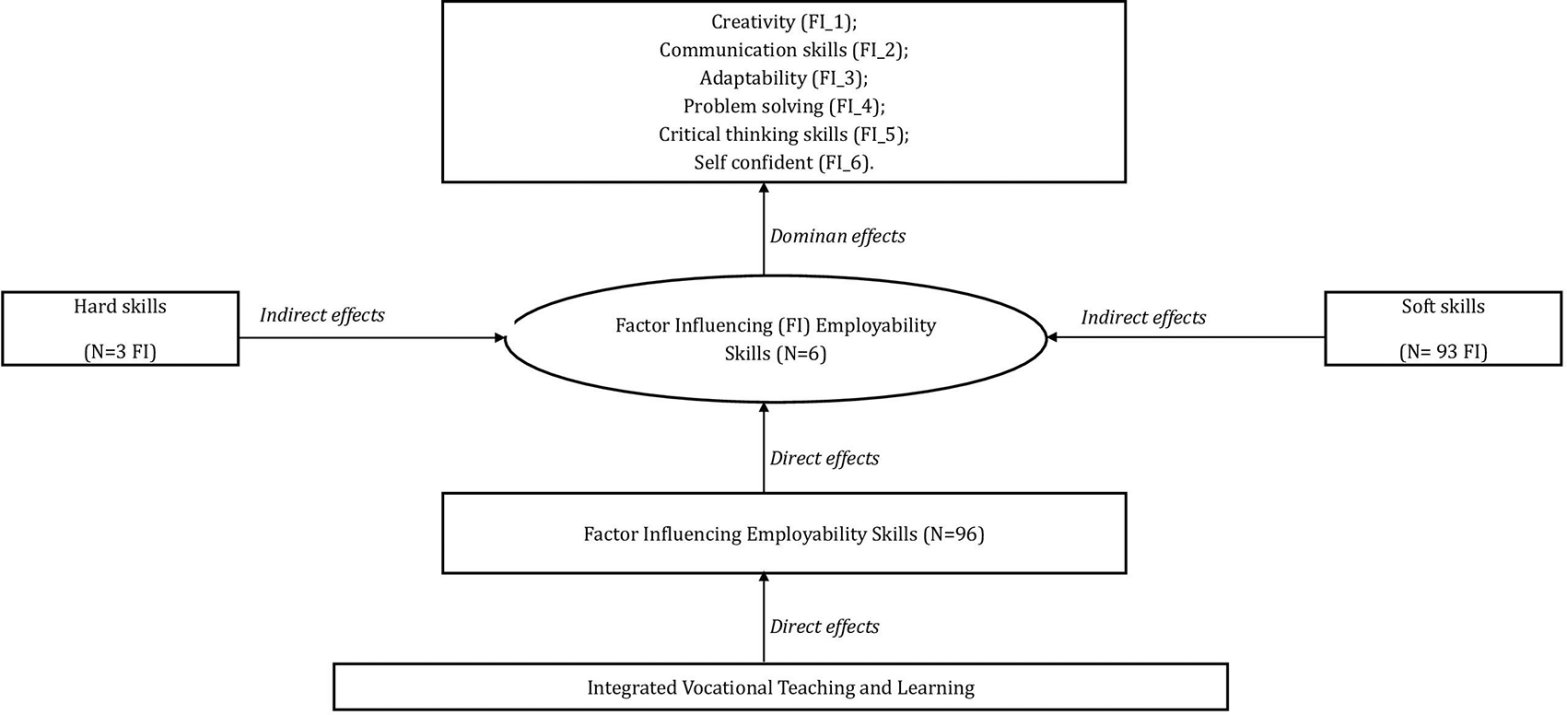
Analysis results of conceptual factor influencing in vocational learning.

Factors that influence employability skills in vocational learning are oriented towards developing individual students’ skills comprehensively. The work skills aspect is important as a reference for researchers to analyze the factors that influence it. Based on these findings, skills, attitudes, and understanding of employability skills become a single unit that influences each other based on hard and soft skills. Student understanding can help complete work well, while skills need students to develop in the workplace with the ability to interact positively with other people, and attitudes determine how to work and determine an individual’s self-orientation to develop, grow, and have a good future. The aspects of vocational school students’ work readiness and their relevance in the integration of vocational learning were reconstructed based on the literature review presented in Figure 10.

### Limitation evidence

The limitations in describing the evidence of articles according to the needs of the world of work in each country vary. It is important to further examine the factors that determine the success of employability skills in each article. There is a need to examine in more detail the suitability of determining factors for the integration of learning in vocational schools. The dominant factors are based on information in journals that needs to be directly linked to the needs of the current world of work. Limitations in determining the inclusion and exclusion criteria for research using PESTLE analysis. Not all the components were met. Political factors determining the policies of each country are different, so determining the influencing factors for employability skills depends on the needs of graduates who are ready to work. The economic factors observed in terms of unemployment rates in each region are also different. Social factors affecting career attitudes are needed. Technological factors related to literacy and technological awareness. Legal factors related to the basis of policies regulated by citizens. Environmental factors related to the characteristics of different vocational schools.

### Limitation review

Limitations in journal searches in terms of bias reduction to minimize errors that occur when selecting articles that are eligible for review. Search limitations by determining keywords cause limitations in the results of articles found by researchers. The keywords used for employability skills are different from employability attitudes, and vocational school students also have meanings that are close to vocational high school students. This is a limitation of this study, as it has not been able to accommodate articles with relevant keyword meanings. Based only on written keywords. Therefore, researchers were careful to reduce the limitations of the review to reduce bias by involving 3 authors in reviewing the article by three authors. The inclusion and exclusion criteria were determined using the PESTLE formula so that it was more directed and focused on the research objectives. Furthermore, the database obtained is not only one source of Google Scholar but also Scopus and others.

### Analysis results of conceptual factor influencing in vocational learning

The results of the reconstruction of factors that directly influence employability skills, based on the literature review, show that there are dominant determining factors. Vocational learning integration considers 96 general factors that must be considered in vocational graduate-oriented learning management ( Figure 10). The indirect impact of students’ hard and soft skills on the preparation of vocational school graduates who are career-ready and globally competitive has a dominant role in the implementation of learning. The dominant factor lies in students’ soft skills, which are relevant to 21st-century skills in the form of creative thinking skills, communication skills, adaptation, critical thinking, and problem-solving skills.

Students’ ability to prepare for the world of work is still very limited and rarely developed in integrated learning in the Business World of Industry (DUDI); therefore, vocational graduates are not ready to be deployed with maximum abilities. The ability to work is the main ability of vocational school students to solve factual problems, which is still worrying. Continuity of learning expects graduates to be ready to work, but currently, it is still oriented towards fulfilling the curriculum and other attributes. ^12, 13^

Figure 10 shows the six indicators of employability that have the most direct influence on students: creativity (FI_1), communication skills (FI_2), adaptability (FI_3), problem-solving (FI_4), critical thinking skills (FI_5), and self-confidence (FI_6). Vocational learning in fostering creativity (FI_1) not only provides memorization of theories and concepts but also contributes to training students’ opportunities to think flexibly, and originally, and be able to provide creative ideas in solving problems. Creative thinking refers to thinking that can prove and produce creative work. ^14– 16^

The management of vocational learning in developing communication skills (FI_2) as a form of student interaction to explore information and relationships between people is highly considered in the world of work. ^17, 18^ The learning process that emphasizes students’ active communication is realized not only by taking notes and listening, but also by developing knowledge and creating active discussions in vocational learning. Communication is characterized by skills in behavior, language, and skills in presenting scientific performance.

Vocational learning has great potential for fostering adaptability skills (FI_3) in the environment. The ability to adapt to the environment as a form of comfort and habits, and being able to adapt to the work environment, enables students to carry out work activities enthusiastically and with the target. ^19, 20^ Knowledge and skills in studying phenomena can improve students’ habits of responding and interacting directly. Students' adjustment to learning new things has an accurate perception of reality, no anxiety or pressure, and they consider themselves to have great potential. Adaptation to new habits can adapt to the environment in the form of changes in behavior or thought patterns. Adaptation involves repeated and permanent behavior over time. This provides vocational graduates ready to face challenges in the world of work. The formation of new habits requires time, certain conditions, and situations; therefore, the vocational learning process requires flexible learning methods and patterns to adapt to the development of students' interests and talents.

Findings on the problem-solving indicator (FI_4) can develop personal competence, which requires strong problem-solving abilities. Problem-solving-oriented learning can provide understanding by stimulating students to pay attention to, study, and think about solving problems. Students' habits in problem-solving activities are an alternative solution to career success, ^21– 23^ Vocational learning must involve the students' full activity in solving problems based on their abilities. Vocational learning should be carried out through scientific performance to develop the ability to think, work, and behave scientifically as an important aspect of life skills.

Critical thinking (FI_5) plays an important role in building human resources and contributes to the progress and development of students' competencies in the world of work. ^24– 26^ Critical thinking is a form of thinking that has reason and is reflective of decision-making. Students' habits in vocational learning activities are required to develop knowledge in the form of facts and concepts relevant to life. To ensure that the critical thinking process is clearly directed, learning management that provides students with opportunities to recognize problems, find solutions, gather information, interpret ideas, evaluate, and analyze is needed.

The self-confidence indicator (FI_6) determines a person's career. Having good self-confidence improves student performance. Motivation for branding oneself as worthy and capable of completing work well is a form of self-management. ^27, 28^ Students' lack of self-confidence results in fear, passivity, lack of motivation to learn, and difficulty in finding their identity. Vocational learning strengthens self-confidence through students' opportunities to argue, provide ideas, and be active in discussions, so that they can develop their potential.

Indicators of factors determining employability skills can be improved through the implementation of learning models that train students in critical thinking skills, communication skills, and problem-solving, which will realize student creativity. The learning process involves adapting to the learning environment with confidence. One vocational project learning approach applied science studies, which can be applied to the world of work and industry through environmental interventions. ^29, 30^ Global challenges require learning patterns that foster high-level thinking and employability. The low level of students' thinking skills is triggered by teachers who consider thinking a process that is only carried out individually. ^5^ In the learning process, teachers do not give students the habit of thinking; therefore, ways to improve students' creative thinking and employability skills are still limited. ^31– 33^

Based on the research results, vocational learning with an environmental orientation can stimulate science process skill activities, student appreciation, and learning achievement. ^34^ The ability to understand science and the environment through issue strategies in society can foster an attitude of environmental concern and maintain traditions and community culture. Vocational learning is important to achieve creative education graduates who can compete in the global era with employability skills and technological capabilities ^35, 36^ while still upholding cultural traditions. ^37^

Consideration of the factors that influence students' employability skills in vocational learning must focus on meaningful environmentally oriented learning management. Students' habit of thinking creatively in solving problems is one of the determining factors of work skills. The employability skills research trend over the last five years (2020-2024) illustrates very clearly that students' needs are not just knowledge but also skills and attitudes. The readiness of vocational graduates, who are reliable, professional, and globally competitive, requires learning management by considering contextual models of problem orientation to train students' thinking habits.

## Conclusions

The research results show that the employability skills of vocational students have experienced a fluctuating trend over the last five years (2020-2024) have experienced a fluctuating trend. The problem of increasingly complex job skills needs is that we must be able to adapt to developments over time and the flow of globalization, so that the needs of vocational graduates are also increasingly changing. Based on a systematic review, 96 factors influenced work skills. There are 6 (six) most dominant determinants of employability skills: creativity (FI_1), communication skills (FI_2), adaptability (FI_3), problem-solving (FI_4), critical thinking skills (FI_5), and self-confidence (FI_6). Vocational learning requires management and meaningful learning. Internationally, the most dominant determinants of work skills are self-confidence, adaptability, and communication skills, whereas in Indonesia, the dominant work skill factors are communication skills and creativity. The research contribution to the employability skills factor is the consideration of six determining factors that can be used as qualifications for graduates in contextual and meaningful vocational learning.

### Ethics and consent

Ethical approval and consent were not required **.**

## Data Availability

No data associated with this article. Figshare: Employability skills article findings and PRISMA analysis results. https://doi.org/10.6084/m9.figshare.29143883.v1. ^38– 73^ Figshare: PRISMA flowchart for employability skills, https://doi.org/10.6084/m9.figshare.29209745.v1. ^38^ This project contains data employability skills article findings and PRISMA analysis results. Data are available under the terms of the Creative Commons Attribution 4.0 International license (CC-BY 4.0).
