# Supplementary material for: Factors that influence the employability skills of vocational school students: A Systematic Review
Source: F1000Res. 2026 Jul 2;14:586. Originally published 2025 Jun 16. [Version 2] doi: 10.12688/f1000research.164629.2 (PMC12344406; doi:10.12688/f1000research.164629.2)
Supplement: Peer review history

# Reviewer report: Reviewer response for version 2

**DOI:** 10.5256/f1000research.204735.r498058

**Published:** 2026-07-10

**Authors:** Samira Dishari

**Affiliations:** 1 Americam university of Middle East, Eqaila, Kuwait

I have now reviewed the revised version and agree that the author has made substantial improvements to the methodology, discussion ,limitations, and overall language of the manuscript.

Are the rationale for, and objectives of, the Systematic Review clearly stated?

Partly

Is the statistical analysis and its interpretation appropriate?

Partly

If this is a Living Systematic Review, is the ‘living’ method appropriate and is the search schedule clearly defined and justified? (‘Living Systematic Review’ or a variation of this term should be included in the title.)

Yes

Are sufficient details of the methods and analysis provided to allow replication by others?

Yes

Are the conclusions drawn adequately supported by the results presented in the review?

Partly

Reviewer Expertise:

NA

I confirm that I have read this submission and believe that I have an appropriate level of expertise to confirm that it is of an acceptable scientific standard.

# Reviewer report: Reviewer response for version 1

**DOI:** 10.5256/f1000research.181169.r396160

**Published:** 2025-08-12

**Authors:** Samira Dishari

**Affiliations:** 1 Americam university of Middle East, Eqaila, Kuwait

The article “ *Factors that influence the employability skills of vocational school students: A Systematic Review”* by Puji Hartini et al., provides useful insights into employability factors in vocational education, highlighting high unemployment rates among vocational graduates and gaps between education and industry needs. The systematic review methodology, including the PRISMA framework and VOS viewer mapping, is a strength of the paper especially the  good data visualization .The inclusion of 96 influencing factors from 39 studies shows a commendable effort in comprehensive data analysis. Researchers present clear identification of six dominant employability factors: creativity, communication, adaptability, problem-solving, critical thinking, and self-confidence. In addition to comparative analysis between international and Indonesian contexts adds depth and relevance.

 However, it would benefit from clearer research framing, improved academic language, and deeper analytical engagement with the reviewed literature.

 1-Abstract

 The abstract does not clearly articulate the article’s central research question, leaving readers without a focused understanding of the study's primary aim. Additionally, it fails to adequately highlight the key findings derived from the systematic review, particularly the six dominant employability skill factors identified. The abstract would also benefit from a brief statement on the study’s implications and a clear direction for future research, which are currently missing. A more structured and informative abstract would significantly enhance the clarity and impact of the article.

 2-Research Questions – Clarity and Focus

 The research question— *to analyze research trends and the factors that influence employability skills of vocational school students*—is generally clear and relevant, especially in the context of global labor market demands. However, the articulation of the research question could be more explicit and formally stated. The authors present multiple issues (e.g., curriculum problems, skills mismatch, and graduate readiness), which may obscure the central aim. A clearly framed question—perhaps in a standalone paragraph or heading—would enhance focus.

 3-Methodology: The use of tools like PRISMA and VOSviewer is effective, but the results would be more impactful if accompanied by critical synthesis and policy-oriented recommendations

 4- Analysis and Discussion

  The discussion and interpretation of the results present several concerns:

 •           Analysis is largely descriptive; it reports frequency counts but lacks critical synthesis of why certain factors are dominant or how they interact.

 •           The discussion section reiterates earlier results rather than advancing deeper insight into implications for educational policy or practice.

 •           The relationship between the PESTLE framework and findings is not fully explored. It is unclear how political, economic, and environmental factors were practically integrated into the analysis.

 5- Limitations are acknowledged, especially in keyword selection and article inclusion, but the implications of these limitations are underdeveloped.

 6- Language and Writing Quality

 The language used throughout the paper is generally understandable and informative, but there are notable areas where grammar, coherence, and academic tone need improvement:

 •           Redundancies and awkward constructions are common, particularly in the abstract and conclusion. For example:

 “The problem of increasingly complex job skills needs is that we must be able to adapt to developments…” – This is convoluted and would benefit from simplification.

 •           The academic tone is occasionally weakened by informal phrasing and repetitive statements.

 •           Some sentences lack fluency or are overly long, which can hinder comprehension.

**Recommendations:** Deepen the interpretation and critique of findings by integrating theoretical frameworks or models of employability and linking them to broader educational or labor market trends.

**Decision:** With revisions, this article has the potential to offer a strong contribution to the field.

Are the rationale for, and objectives of, the Systematic Review clearly stated?

Partly

Is the statistical analysis and its interpretation appropriate?

Partly

If this is a Living Systematic Review, is the ‘living’ method appropriate and is the search schedule clearly defined and justified? (‘Living Systematic Review’ or a variation of this term should be included in the title.)

Yes

Are sufficient details of the methods and analysis provided to allow replication by others?

Yes

Are the conclusions drawn adequately supported by the results presented in the review?

Partly

Reviewer Expertise:

My areas of expertise include teaching methodologies and curriculum design. In terms of research, I frequently use systematic reviews and scoping reviews as my primary approaches for publication.

I confirm that I have read this submission and believe that I have an appropriate level of expertise to confirm that it is of an acceptable scientific standard, however I have significant reservations, as outlined above.

# Response

**Published:** 2026-01-07

**Authors:** Qori Agussuryani Puji Hartini

**Affiliations:** Department of Natural Science, Universitas Negeri Semarang, Semarang, Central Java, Indonesia

REVIEW 1:

 The article “ *Factors that influence the employability skills of vocational school students: A Systematic Review”* by Puji Hartini et al., provides useful insights into employability factors in vocational education, highlighting high unemployment rates among vocational graduates and gaps between education and industry needs. The systematic review methodology, including the PRISMA framework and VOS viewer mapping, is a strength of the paper especially the  good data visualization .The inclusion of 96 influencing factors from 39 studies shows a commendable effort in comprehensive data analysis. Researchers present clear identification of six dominant employability factors: creativity, communication, adaptability, problem-solving, critical thinking, and self-confidence. In addition to comparative analysis between international and Indonesian contexts adds depth and relevance.

 However, it would benefit from clearer research framing, improved academic language, and deeper analytical engagement with the reviewed literature.

 RESPON 1:

**The scientific language complies with standard journal language rules and is supported by accurate data, as shown in Table 2 and Figures 2 to 8.**

 REVIEW 2:

 1-Abstract

 The abstract does not clearly articulate the article’s central research question, leaving readers without a focused understanding of the study's primary aim. Additionally, it fails to adequately highlight the key findings derived from the systematic review, particularly the six dominant employability skill factors identified. The abstract would also benefit from a brief statement on the study’s implications and a clear direction for future research, which are currently missing. A more structured and informative abstract would significantly enhance the clarity and impact of the article.

 RESPON 2:

**The abstract includes a key question on: This study analyzes the research trends and factors that influence vocational high employability.**

**Furthermore, there are contributions, limitations and implications of the research (written: The determining factors of work skills internationally are more dominant in self-confidence, adaptability, and communication skills, while in Indonesia, they are communication skills and creativity. A limitation of the data search process is the use of incomplete keywords. The contribution of this study is that six determining factors were used as considerations for the qualifications of student graduates in contextual and meaningful vocational learning).**

 REVIEW 3:

 2-Research Questions – Clarity and Focus

 The research question— *to analyze research trends and the factors that influence employability skills of vocational school students*—is generally clear and relevant, especially in the context of global labor market demands. However, the articulation of the research question could be more explicit and formally stated. The authors present multiple issues (e.g., curriculum problems, skills mismatch, and graduate readiness), which may obscure the central aim. A clearly framed question—perhaps in a standalone paragraph or heading—would enhance focus.

 RESPON 3:

**In the final paragraph of the introduction, the research question is stated (written: The problem of employability skills is very complex in analyzing dominant factors, so the research objectives are to analyze research trends in the employability skills of vocational school students and factors that influence the employability skills of vocational school students). Next, the issue is presented in the background to reinforce that this problem is important to research.**

 REVIEW 4:

 3-Methodology: The use of tools like PRISMA and VOSviewer is effective, but the results would be more impactful if accompanied by critical synthesis and policy-oriented recommendations.

 RESPON 4:

**Thank you for your review, and the critical analysis of the results from Vosviewer shows that the closer the distance between studies, the stronger the relationship, and the stronger the citations between journals. Vocational education does not fully refer to job skills and is more dominant in technological vocational education training; therefore, further studies are needed on job skill factors.**

 REVIEW 5:

 4- Analysis and Discussion

  The discussion and interpretation of the results present several concerns:

 •           Analysis is largely descriptive; it reports frequency counts but lacks critical synthesis of why certain factors are dominant or how they interact.

 •           The discussion section reiterates earlier results rather than advancing deeper insight into implications for educational policy or practice.

 •           The relationship between the PESTLE framework and findings is not fully explored. It is unclear how political, economic, and environmental factors were practically integrated into the analysis.

 RESPON 5:

- **This dominant factor is determined based on the results of a review of previous research which stated the percentage of skills findings in the last five years (2020-2024) and 14 countries internationally stated six factors influencing employability skills (creativity (FI_1), communication skills (FI_2), adaptability (FI_3), problem-solving (FI_4), critical thinking (FI_5), and self-confidence (FI_6)).**
- **It was added that the implications of educational policies and practices must focus on the demands of work skills currently needed, so that educational practices in realizing the profile of vocational school graduates must have professional skills.**
- **Political, economic and environmental factors have been practically explained a little regarding the support system in developing broader work skills, but within the limits of this research the focus is on work skills in vocational school learning.**

REVIEW 6:

 5- Limitations are acknowledged, especially in keyword selection and article inclusion, but the implications of these limitations are underdeveloped.

 RESPON 6:

**One limitation of the data retrieval process was the use of incomplete keywords. However, the researchers supported this with scientific arguments and previous research data, ensuring the accuracy and validity of the data.**

 REVIEW 7:

 6- Language and Writing Quality

 The language used throughout the paper is generally understandable and informative, but there are notable areas where grammar, coherence, and academic tone need improvement:

 •           Redundancies and awkward constructions are common, particularly in the abstract and conclusion. For example:

 “The problem of increasingly complex job skills needs is that we must be able to adapt to developments…” – This is convoluted and would benefit from simplification.

 •           The academic tone is occasionally weakened by informal phrasing and repetitive statements.

 •           Some sentences lack fluency or are overly long, which can hinder comprehension.

 RESPON 7:

**The need for work skills is increasingly complex and students must be able to adapt to existing global developments. With skills readiness, difficulties and weaknesses in work skills will be overcome.**

**REVIEW 8:**

**Recommendations:** Deepen the interpretation and critique of findings by integrating theoretical frameworks or models of employability and linking them to broader educational or labor market trends.

 RESPON 8:

**Interpretations are added to the criticism of the findings of Vosviewer results which show that it is not only the six factors influencing employability skills, but further than these results is how to package learning management with vocational training.**

**Thank you for the review, with the revision the article will be of better quality and strengthen the scientific results.**

# Reviewer report: Reviewer response for version 1

**DOI:** 10.5256/f1000research.181169.r392708

**Published:** 2025-06-25

**Authors:** Mohd Fauzi Sedon

**Affiliations:** 1 Sultan Idris Education University, Perak, Malaysia

This systematic review examines 39 studies (from 2020–2024) to identify factors influencing the employability skills of vocational high school students. Using the PRISMA approach and databases like Scopus, Google Scholar, and Web of Science, the authors identified six dominant employability skills factors: Creativity, Communication skills, Adaptability, Problem-solving, Critical thinking and Self-confidence. International and Indonesian data were compared, with findings suggesting different dominant skill emphases by region. The study highlights gaps in vocational curricula and calls for more integrated, skills-oriented learning aligned with industry needs.

 Overall, a useful contribution to vocational education research, especially in aligning training with 21st-century workforce demands.

Reviewer Expertise:

Education, Culture and Tradition, Visual Arts, Creative Industry, Green and Sustainability, Social Sciences

I confirm that I have read this submission and believe that I have an appropriate level of expertise to confirm that it is of an acceptable scientific standard.

# Response

**Published:** 2025-06-29

**Authors:** Qori Agussuryani Puji Hartini

**Affiliations:** Department of Natural Science, Universitas Negeri Semarang, Semarang, Central Java, Indonesia

Thank you for your review, which will add to the strengthening of scientific contributions to research in the field of employability skills of vocational school students.
